# New therapeutic targeting of Alzheimer’s disease with the potential use of proline-rich polypeptide complex to modulate an innate immune response - preliminary study

**DOI:** 10.1186/s12974-019-1520-6

**Published:** 2019-07-05

**Authors:** Marta Sochocka, Michał Ochnik, Maciej Sobczyński, Iwona Siemieniec, Beata Orzechowska, Piotr Naporowski, Jerzy Leszek

**Affiliations:** 1 Laboratory of Virology, Department of Immunology of Infectious Diseases, Hirszfeld Institute of Immunology and Experimental Therapy, Polish Academy of Sciences, Weigla 12, 53-114 Wroclaw, Poland; 2 Department of Genomics, Faculty of Biotechnology, University of Wroclaw, Wroclaw, Poland; 3 Laboratory of Medical Microbiology, Hirszfeld Institute of Immunology and Experimental Therapy, Polish Academy of Sciences, Wroclaw, Poland; 4 Department of Psychiatry, Wroclaw Medical University, Wroclaw, Poland

**Keywords:** Impaired innate immunity, Vesicular stomatitis virus (VSV), Resistance to viral infection, Alzheimer’s disease, Proline-rich polypeptide complex (PRP), Cytokine, PBLs

## Abstract

**Background:**

The lack of effective treatment for Alzheimer’s disease (AD) stems mainly from the incomplete understanding of AD causes. Neuroinflammation has emerged as an important component of AD pathology, and a vast number of experimental and clinical data indicated a crucial role for the activation of the innate immune system in disease promotion and symptom progression.

**Methods:**

Clinical examinations of AD patients in a different stage of disease severity in correlation with the measurement of two innate immune reactions, i.e., peripheral blood leukocyte (PBLs) resistance to viral infection (vesicular stomatitis virus, VSV) ex vivo, and cytokines: TNF-α, IFN-γ, IL-1β, and IL-10, production with enzyme-linked immunosorbent assay (ELISA), have been investigated during this preliminary study before and after 4 weeks of oral treatment with dietary supplement proline-rich polypeptide complex (PRP) (120 μg of PRP/day). The potential effect of PRP on the distribution of PBLs’ subpopulations has been specified.

**Results:**

We have found a deficiency in innate immune response in AD patients. It was demonstrated for the first time that the degree of PBLs resistance to VSV infection was closely related to the stage of clinical severity of AD. Our study showed significant differences in cytokine production which pointed that in AD patients innate immune mechanisms are impaired. Administration of PRP to our patients increased innate immune response of PBLs and declined pro- and anti-inflammatory cytokine production, thus subduing the excessively developed inflammatory response, especially among patients with high severity of AD. PRP did not exhibit a pro-proliferative activity. It was showed, however, significant influence of PRP on the distribution of PBLs’ subpopulations.

**Conclusion:**

The findings mentioned above might be crucial in the context of potential application of immunomodulatory therapy in AD patients and indicated PRP as a potential target for future treatments in neuroinflammatory diseases like AD.

**Electronic supplementary material:**

The online version of this article (10.1186/s12974-019-1520-6) contains supplementary material, which is available to authorized users.

## Background

Alzheimer’s disease (AD) is the most common type of dementia that affects millions of people around the world. Age is one of the most important non-modifiable risk factor of AD; thus, the disease primarily affects the elderly [1]. It should be noticed however that neurodegenerative changes can commence at any age [2]. Aging is a complex process, depending on many lifestyle and environmental factors and genetic and epigenetic events occurring in various types of cells and tissues throughout life [3]. Significant feature of organismal aging is an aging of the immune system called “immunosenescence” that in consequence leads to both an impaired adaptive and innate immune response [4]. During aging of the innate immune system, inflammatory responses are dysregulated, which may lead to an exertion of pro-inflammatory milieu in humans. In the case of such chronic inflammation, an innate immune activation may be impaired, especially in response to pathogens [5]. The consequences of failure in the innate immune response have potential implications for age-associated chronic inflammatory conditions, including AD [6, 7].

Peripheral blood leukocytes (PBLs) resistance to viral infection was found to be an indicator of the innate immune system condition. The strongest PBLs resistance to viral infection was observed ex vivo, and it is maintained in vivo as one of the innate immunity reactions in the healthy organism [8, 9]. To evaluate the level of PBLs resistance, a test based on vesicular stomatitis virus (VSV) replication in freshly isolated PBLs (ex vivo) was designed [8]. The sensitivity of PBLs to VSV infection is an indicator of the condition of the immune system. Lack of VSV replication, 0–1 log TCID_50_ (tissue culture infectious dose), indicated a high level of innate immunity which was described as a complete resistance. A low level of VSV replication (2–3 log) was denoted as a partial resistance. A high level (≥ 4 log) indicated deficiency in innate immunity. As previously reported, an innate antiviral immune response of PBLs is age-related and individually differentiated [10]. It was also shown that PBLs’ sensitivity to VSV infection (deficiency in innate immunity) is very high among patients with frequent incidence of herpes labialis upper respiratory tract infections [8], as well as in cancer patients and patients with AD [10]. The target cells of VSV replication are monocytes and monocyte-derived dendritic cells (MDDCs). Virus induces strong activation of innate mechanisms with upregulated expression of retinoic acid-inducible gene I (RIG-I)-like receptors (RLRs) and IFN-stimulated genes (ISGs), as well as stimulation of several cytokine and chemokine production such as TNF-α, IFN-α, and IP-10 [11]. It was shown previously that the whole PBLs are the best model for the assessment of the innate immune system condition [12]. Although VSV infects only monocytes [11], the role of the other immune cells in response to viral infection cannot be diminished. The most important feature of this mechanism is cytokine networking. Cytokines produced by one type of innate immune cells initiate the cascade of events to restrict viral infection, and in a consequence, they also influence the other immune cells. Therefore, among the whole population of leukocytes, the regulation mechanism exists and the degree of resistance measured in the whole PBLs is the most informative and repeatable [12]. Several therapeutics with potential immunoregulatory activity was studied, such as donepezil, extract of *Gingko biloba*, and flavones from *Scutellaria baicalensis*. Their strong ability to increase PBLs resistance to VSV infection (to increase innate immunity) of healthy donors as well as influence on cytokine production has been established already [13–15].

In relation to a still growing number of patients with AD, understanding the immune-related mechanisms of the disease and searching for compounds that have potency to modulate immunological deficits in this group of patients seem to be fully justified. AD belongs to the group of diseases that cause the highest mortality in people all over the world; therefore, developing more effective treatments that could increase the expectancy and quality of life of afflicted is highly recommended [16]. We expected that positive results on innate immune reactions in AD patients may be obtained with treatment with proline-rich polypeptide complex (PRP) isolated from bovine colostrum, which is considered as a promising nutraceutical intended to boost an immune system [17, 18]. PRP isolated from ovine colostrum has been shown to influence the kinetics of VSV replication and possess immunoregulatory properties, including effects on humoral and cellular immune responses; shows regulatory activity in Th1 and Th2 cytokine induction; and has the ability to inhibit the overproduction of reactive oxygen species (ROS) and nitric oxide (NO) [19–21]. This suggests a possible positive influence on AD patients. The positive impact of PRP on cognitive functions has been shown in several clinical trials [21, 22]. To date, no in vivo study investigating the influence of PRP treatment on innate immune reactions in AD patients has been reported.

In our study, two reactions of innate immunity were examined, i.e., ex vivo PBLs resistance to viral infection, and cytokine production by PBLs from AD patients before and after PRP treatment. This paper presents for the first time very interesting results that support the hypothesis of impaired immune response of PBLs (deficiency in innate immunity) among AD patients and indicate PRP as a potent agent that can modulate immunological responses in this group.

## Materials and methods

### Study design

A before-after study was conducted with participation of AD patients to evaluate the impact of proline-rich polypeptide complex (PRP) on innate immune response in AD patients.

### Patients and blood samples

The study comprised of 25 participants (16 females, 9 males) aged 43–79 years. Participants include patients under the care of the Department of Psychiatry of the Medical University in Wroclaw, Poland. Patients did not receive any anti-dementia and other drugs during the study as well as any other immunomodulators. Among the patients, no infectious diseases occurred in the 3 period months before the inclusion to the study Peripheral venous blood was obtained from all subjects and collected in tubes containing anticoagulant EDTA for future investigations of two mechanisms of innate immunity. Additional blood samples were collected for C-reactive protein (CRP) measurement before and after PRP treatment.

### Clinical examination

The baseline examination included psychiatric and neurological examinations, as well as laboratory tests, electroencephalographic (EEG) examinations, and computer tomography (CT) or magnetic resonance imaging (MRI) structural studies. The Mini-Mental State Examination (MMSE) was used for the screening of dementia. All patients enrolled in the study met DSM V and NINCDA-ADRDA criteria for probable AD dementia. A diagnosis of AD is made when specific symptoms are present and by making sure other causes of dementia are not present, including anemia, brain tumor, chronic infection, intoxication from medication, severe depression, stroke, thyroid disease, and vitamin deficiencies. CT and MRI of the brain were performed as well to look for other causes of dementia, such as brain tumor or stroke. Semistructered interview with the patient and informant, physical exam, evaluation of neurological status, and psychiatric exam were obtained. Vital signs and blood screening labs (hematology, chemistry panel, urinalysis, B12, TSH) were collected. Exclusion criteria were as follows*:* patients older than 90 years; any significant neurological disease such as Parkinson’s disease, multi-infarct dementia, Huntington disease, normal pressure hydrocephalus, brain tumor, progressive supranuclear palsy, seizure disorder, subdural hematoma, and multiple sclerosis; or history of significant head trauma followed by persistent neurologic defaults or known structural brain abnormalities; MRI scan with evidence of infection, infarction, or other focal lesions; subjects with multiple lacunes or lacunes in a critical memory structure; psychiatric disorder/psychotic features: major depression, bipolar disorder, agitation or behavioral problems within the last 3 months, and history of schizophrenia; alcohol abuse, history of alcohol or substance abuse or dependence within the past 2 years; any significant systemic illness or unstable medical condition; clinically significant abnormalities in B12, RPR, or TSH; and current use of specific psychoactive medications (e.g., certain antidepressants, neuroleptics, chronic anxiolytics, or sedative hypnotics). Patients were excluded if they did not agree to respond to the test questions and/or if they had life-threatening diseases other than AD.

### Proline-rich polypeptide complex (PRP) treatment

Twenty-five AD patients were treated with PRP preparation (Recognizin prp, dietary supplement, provided by Le Loch Healthcare, Poland). Each patient took 1 tablet that contains 120 μg of PRP every day for 4 weeks (oral administration). After the end of the study period, final results for 20 patients, before and after 4 weeks of PRP treatment, were completely collected and analyzed. Five patients were excluded due to incidents not related to the study that is serious injuries or diseases (e.g., ischemic stroke) that occurred during the study.

### Virus and cell line

A wild-type Indiana VSV (vesicular stomatitis virus, *Rhabdoviridae*) serotype was originally obtained from Dr. C. Buckler (National Institutes of Health, Bethesda, MD, USA). VSV was grown and titrated in L_929_ cells. The titer was expressed with reference to the TCID_50_ (tissue culture infectious dose) value, based on the cytopathic effect caused by this virus in approximately 50% of infected cells.

L_929_ (ATCC CCL1), a murine fibroblast-like cell line, was maintained in complete RPMI 1640 medium (IIET, Wroclaw, Poland) with antibiotics (100 U/mL penicillin and 100 μg/mL streptomycin), 2 mM L-glutamine, and 2% fetal bovine serum (FBS) (all from Sigma-Aldrich, USA).

### Isolation of peripheral blood leukocytes (PBLs)

PBLs were isolated according to a standard protocol from peripheral blood by gradient centrifugation in Gradisol G (Aqua-Med, Łódź, Poland) and maintained in RPMI 1640 medium (IIET, Wroclaw, Poland) with antibiotics (100 U/mL penicillin and 100 μg/mL streptomycin), 2 mM L-glutamine, and 2% FBS (all from Sigma-Aldrich, USA).

### Determination of resistance/level of innate immunity of PBLs

Resistance/innate immunity was determined by in vitro infection of leukocytes (1 × 10^6^ cells/ml) with a VSV dose of 100 TCID_50_. After 40 min of adsorption at room temperature (rt), the virus was washed out three times with 5 ml of RPMI medium and the cells were suspended in 1 ml of RPMI medium with 2% FBS. A sample of the infected cells was kept at 4 °C and served as a control of the starting level of the virus. The rest of the cells were incubated at 37 °C, and samples of medium above the infected cells were collected after 24 h and titrated in L_929_ cells. The titer of virus was expressed in TCID_50_. Resistance of PBLs to VSV infection was assessed according to a method described earlier [23]; a VSV titer ≥ 4 log TCID_50_ was considered as a lack of resistance (deficiency in innate immunity), a titer of 2–3 log indicated partial resistance, and a titer of 0–1 log indicated complete resistance to VSV infection (high level of innate immunity).

### Cytokine measurement

The levels of IL-10, IL-1β, IFN-γ, and TNF-α in supernatants from uninfected and VSV-infected leukocytes were detected using enzyme-linked immunosorbent assay (BD OptEIA TM human IL-10, IL-1β, IFN-γ, or TNF-α enzyme-linked immunosorbent assay (ELISA) set, BD Biosciences). The optical density was measured at 450 nm with *λ* correction of 570 nm using a Multiskan RC spectrophotometric reader (Thermo Labsystems, USA). Cytokine concentrations were expressed in picograms per ml.

### MTT assay for cell viability and proliferation

The MTT assay is the enzyme-based method, which use the 3-(4,5-dimethylthiazol-2-yl)-2,5-diphenyltetrazolium bromide as a reductive coloring agent. PBLs (1 × 10^6^ cells/ml) were cultured in 96-well plates in the presence of PRP (120 μg/ml) for 48 h at 37 °C in a humidified atmosphere with 5% CO_2_. After 48 h, 20 μL of MTT solution (5 mg/ml) (Promega, Madison, WI, USA) was added to each well, and the microplates were kept at 37 °C/5% CO_2_ for 3 h. Then, the solubilization/stop solution containing SDS-HCl (10% SDS, 0.01 M HCl) (100 μL/well) was added, and the absorbance values of the wells were measured at 570 nm using a 96-well plate reader (Multiskan RC spectrophotometer, Thermo Labsystems, Waltham, MA, USA).

### Analysis of distribution of PBLs’ subpopulations

The whole blood culture (WBC) was established in a 24-well flat bottom (Costar, Cambridge, USA). Peripheral venous blood was diluted 1/5 with RPMI medium with 2% FBS. Next, diluted blood was treated with PRP (120 μg/ml) or medium only and incubated at 37 °C/5% CO_2_. After 24 h of incubation, analysis of distribution of PBLs’ subpopulations was performed with a hematology analyzer Mythic18 (Orphee, Cormay).

### 1D-SDS-PAGE electrophoresis

One-dimensional SDS-PAGE electrophoresis described by Laemmli [24] was performed for proline-rich polypeptide complex (PRP) separation and estimation of protein molecular weights. Suspension of 30 mg PRP in sample buffer (1 M Tris-HCl, 2% SDS, 10% glycerol, 5% β-mercaptoethanol, bromophenol blue) was boiled for 5 min and loaded onto 12.5% polyacrylamide gel. Amersham LMW-protein marker was provided by GE Healthcare. Electrophoresis was performed with using Biometra Minigel-Twin. Proteins were stained with Coomassie brilliant blue G-250/R-250 (Serva) and visualized by using Bio-Rad Gel Doc™ XR+.

### Statistical analysis

Relation between PBLs resistance (innate immunity), MMSE, cytokine levels, and therapeutic effect after 4 weeks of PRP treatment was tested with linear contrast in ANOVA scheme and the Student *t* test for paired groups. All measures was transformed to logarithmic scale at natural base, i.e., *x´* = ln *x*. Effect of PRP treatment for *i*th patient was described as ${\mathrm{ratio}}_i=\ln \frac{{\mathrm{after}}_i}{{\mathrm{before}}_i}$. Expected value of the ratio (i.e., mean value for a group) was calculated as $m={e}^{\frac{1}{n}{\sum}_i^n{\mathrm{ratio}}_i}$, where *e* ≈ 2.7183 is the base of natural logarithms. As a measure of effect size *r*_effect size_, correlation statistic was used, where *r*_effect size_ = $\sqrt{\frac{F_{\mathrm{contrast}}}{F_{\mathrm{contrast}}+{df}_{\mathrm{within}}}}$ and *df*_within_ are degrees of freedom within groups; *F*_contrast_ has one degree of freedom in nominator. Statistic *r*_effect size_ is interpreted as adjusted correlation between two variables (dependent and independent) after one adjusted for both any non-contrast between-group variation and within-group variation and *r*_effect size_ *ϵ* [0,1]. All tested hypotheses were formulated a priori, during the study design. We avoided performing too many and unnecessary statistical comparisons. In case of the effect of PRP treatment, null hypothesis H0 for cytokines was *H*0 : *m* ≥ 1 opposite the alternative *H*1 : *m* < 1, where *m* is defined above. We expect that PRP will decrease the inflammatory response of PBLs so the levels of the investigated cytokines also decrease. Percent of change after treatment was calculated as Change (%) = *m* × 100 − 100. Influence of PRP treatment on PBLs proliferation was reported as the mean of 5 means, where every single mean was computed based on *n* = 8 independent measurements (*N* = 40). ANOVA for PRP treatment after 24 h according to PBLs distribution in randomized block design (blood sample/donor as block) was used. Changes in number of cells were a response, i.e., difference between PRP treatment after 24 h and without PRP treatment after 24 h (main effect: type of cells). Sum of squares for the main effect was divided into two orthogonal contrasts: (c1) difference between mean changes in the population of leukocytes and monocytes and (c2) difference between leukocytes and monocytes (pooled) and granulocytes. Homogeneity of variances was tested with Fligner-Killeen test. Power of the one-sided *T* test, as a probability that the test will reject the null hypothesis if the cytokine level decreases at least − 50%, was also calculated at *α* = 0.05.

## Results

### Study group

Table 1 contains characteristics of the patients included in the study and assessment of PRP treatment on innate immune response of PBLs. There were 13 females and 7 males among 20 participants of the study. MMSE score was evaluated (median 19), and patients were classified as mild AD (5 subjects; 25%), moderate AD (7 subjects; 35%), and serious AD (8 subjects; 40%) based on DSM V criteria. Body mass index (BMI) was also estimated and median was 22.5 (normal range 18.5–24.9). The correct BMI range is important seeing that an excess of adipose tissue constitutes an additional source of inflammatory mediators. Moreover, serum levels of CRP were estimated before and after PRP treatment. Among the subjects, 17 showed a correct CRP level in the range of 0–5 mg/l and 3 patients were above the normal range before the study; however, elevated CRP values were small and patients were assessed as healthy without chronic diseases. After the end of the study, CRP of 19 patients was in the normal range, one was slightly elevated.

**Table 1:** Clinical characteristics of the included patients

Variable	Min	Q1	Median	Q3	Max
Age	43	65.75	68.5	74	79
BMI	18.5	20.75	22.5	23	25
CRP before	0.30	1.00	1.16	2.15	12.90
CRP after	0.31	1.00	1.33	1.98	14.92
CRP before-after	−11.90	−0.18	0.01	0.48	13.69
MMSE	12	14	19	22	23
MoCA 7.2	3	10.75	14	16.25	23
DSM V classification					
Mild		Moderate		Serious	
* n*	%	*n*	%	*n*	%
5	25	7	35	8	40
Gender	*n*	%	Age^*^	BMI^*^	MMSE^*^
Men	7	35	69	22	18
Women	13	65	68	23	19

Q1, Q3—1st and 3rd quartile; ^*^median

### Impaired innate immune response of PBLs of Alzheimer’s disease patients correlates with the clinical severity of the disease

**Fig. 1:**
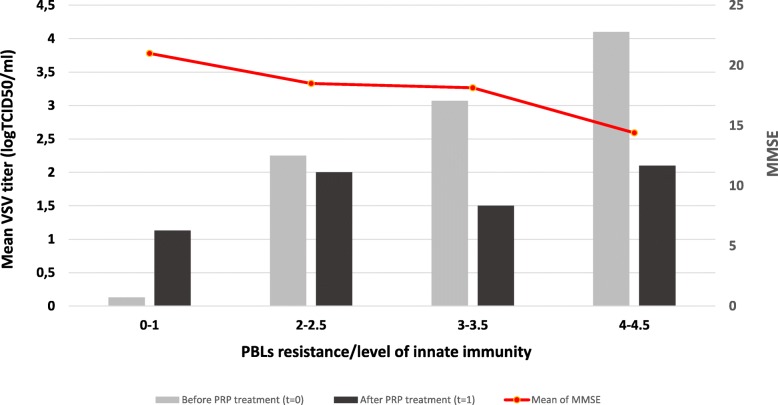
Mean levels of PBLs resistance/level of innate immunity before (*t* = 0) and after PRP treatment (*t* = 1) grouped by innate immunity/PBLs resistance at *t* = 0. Additionally means of MMSE score at *t* = 0 in all groups are presented. Freshly isolated PBLs (1 × 10^6^ /ml) from blood samples of AD patients before and after PRP treatment were infected with VSV (10^2^ TCID_50_/ml). After 40 min of VSV adsorption at RT cells were washed with culture medium and incubated at 37 °C for 24 h. Samples of medium above cultured PBLs were next collected and titrated in L_929_ cells. Red curve represents mean of MMSE score of AD patients. For statistical analysis see description of Table 2

To determine the resistance/innate immunity of PBLs obtained from AD patients ex vivo to VSV infection, the VSV titers were assessed in the collected supernatants after 24 h of leukocyte incubation. We observed significant differences in PBLs resistance among AD patients; however, as showed in Table 2, most of them were characterized with partial or deficient PBLs resistance to viral infection (2–3 log or ≥ 4 log). Results are presented in Fig. 1.

**Table 2:** Means and standard deviations (SD) of MMSE score in AD patients (*n* = 20) in relation to the innate immunity measured with PBLs resistance to viral infection (viral titer express in TCID_50_) at *t* = 0. ANOVA linear contrast analysis was performed to investigate the relationship between the innate immunity of PBLs (range) and effect of PRP (left table) and MMSE score (right table). In addition, the mean of the innate immunity of PBLs at *t* = 0 (before PRP treatment) and *t* = 1 (after PRP treatment) and differences between *t* = 1 and *t* = 0 are shown

PBLs resistance/level of innate immunity				MMSE^**^		
Range *t* = 0	Mean *t* = 0	Mean *t* = 1	Mean difference	Mean	SD	*n*
0–1^*^	0.13	1.13	1.00	21.00	1.83	4
2–2.5	2.25	2.00	− 0.25	18.50	4.73	4
3–3.5	3.07	1.50	− 1.57	18.14	3.72	7
4–4.5	4.10	2.10	− 2.00	14.40	2.79	5
*F*_contrast_ = 12.91, *df* = 1;16, *p* = 0.0024 *r*_effect size_ = 0.66 H_0_: mean difference is the same in all groups H_1_: linear relation between PRP effect and level of innate immunity				*F*_contrast_ = 7.67, *df* = 1;16, *p* = 0.0136 *r*_effect size_ = 0.56 H_0_: mean MMSE is the same in all groups H_1_: linear relation between mean MMSE and level of innate immunity		

*PBLs with complete resistance to viral infection, high level of innate immunity

**Measured at the time of inclusion into the project

*t* = 0 before PRP treatment

*t* = 1 after PRP treatment

Table 2 presents all means and standard deviations for MMSE in four groups of patients in relation to the PBLs resistance (level of innate immunity) measured with TCID_50_. Based on the results shown in Table 2, we conclude that there is a clear and strong relation between innate immunity of PBLs and MMSE score in AD patients. Stronger innate immunity of PBLs correlated with a higher score of MMSE and better mental status of the patients, and vice versa. We can see that the mean of the MMSE score in patients with very good innate immunity/complete PBLs resistance to VSV infection (0–1 log) is 21 points, whereas the mean of MMSE in patients with very low/deficiency of PBLs resistance/innate immunity (≥ 4 log) is only 14.4 points which is defined as a serious stage of AD (according to the DSM V criteria). Relations described above are not accidental (*F*_contrast_ = 7.67, *df* = 1;16; *p* = 0.0136). Statistic *r*_effect size_ = 0.56 is a true correlation between the level of the innate immunity of PBLs and the mean of the MMSE after we adjusted for both non-contrast between-group variation and within-group variation. This effect size is interpreted as medium on Cohen’s statistical effect size scale.

### PRP treatment enhances resistance/innate immunity of PBLs of AD patients

After 4 weeks of PRP treatment, PBLs of all patients were isolated and divided into groups—cultured uninfected and infected with VSV. Next, VSV titer was assessed in the collected supernatants after 24 h of leukocyte incubation. Based on the results from Table 2, we can conclude that after PRP treatment the resistance/level of innate immunity of PBLs from AD patients (*F*_contrast_ = 12.91, *df* = 1;16, *p* = 0.0024) increased. This effect was more evident among patients with very low resistance/innate immunity of PBLs (4–4.5 at *t* = 0, mean difference = − 2) and less marked in patients with partial resistance/innate immunity of PBLs (2–2.5 score at *t* = 0; mean difference = − 0.25). This indicates that PRP treatment had a potency to increase the resistance/level of innate immunity of PBLs and these benefits were most meaningful among patients with high severity of AD (moderate and serious AD according to DSM V criteria). The effect was more than medium on Cohen’s statistical effect size scale, i.e., *r*_effect size_ = 0.66. This statistic is correlation coefficient between the effect of 4 weeks treatment with PRP on the level of innate immunity of PBLs (*t* = 1) and the level of innate immunity of PBLs before treatment (*t* = 0) after adjusting between-group variation sources and within-group variation sources not linked to this relation. In the case of 15 of 20 patients, an increase of resistance/innate immunity of PBLs was observed, and it was 75% with CI95 (52.8%; 88.7%). These results confirmed that in patients whom PRP treatment had a positive impact on innate immunity of PBLs (increase PBLs resistance to VSV infection), the effect is higher than 50%. All results are presented on Fig. 1.

### Cytokine production by PBLs differs among AD patients

**Fig. 2:**
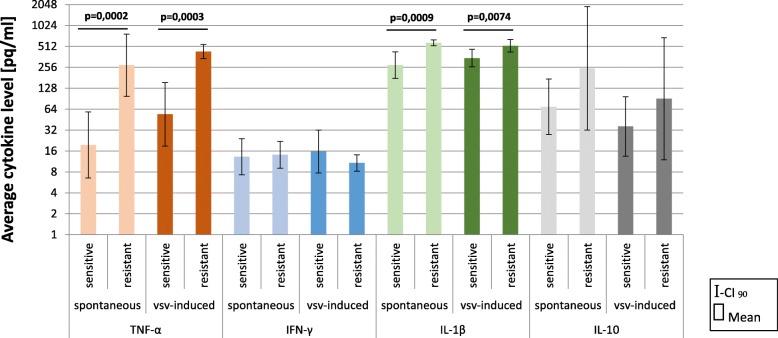
Cytokine profile of PBLs of AD patients before PRP treatment (*t* = 0). Changes in spontaneous and VSV-induced cytokine production by PBLs depending on the PBLs resistance/level of innate immunity. Freshly isolated PBLs (1 × 10^6^ /ml) from blood samples of AD patients before PRP treatment were divided in two groups. One was cultured uninfected and second was infected with VSV (10^2^ TCID_50_/ml). After 40 min of VSV adsorption at RT, PBLs were washed with culture medium. All cells were next incubated at 37 °C for 24 h. Samples of medium above cultured uninfected (spontaneous cytokine production) and VSV-infected PBLs were collected for cytokine measurement with ELISA. Results have been analyzed with the Student *t* test for two groups on log-transformed data

Next, we examined the cytokine profile (TNF-α, IFN-γ, IL-1β, and IL-10) produced by PBLs of AD patients with different levels of resistance/innate immunity. PBLs from all subjects were divided into two groups, with complete resistance (VSV titer 0–1 log) and partial or lack of resistance (VSV titer ≥ 2 log). We noticed statistically significant changes in the concentration of TNF-α and IL-1β between these groups in spontaneous and VSV-induced cytokine production. The most significant effect was observed for spontaneous TNF-α production. Mean level of TNF-α among PBLs with complete resistance was 274.06 pq/ml, whereas in those with partial or lack of resistance it was 19.56 pq/ml, about 14 times more. The effect was more than medium on Cohen’s statistical effect size scale, i.e., *r*_effect size_ = 0.592. Furthermore, 2 times more in spontaneous IL-1β production was detected among PBLs with complete resistance compare to PBLs with partial or lack of resistance. The effect size was medium on Cohen’s statistical effect size scale for IL-1β, i.e., *r*_effect size_ = 0.458. Interestingly, spontaneous IL-10 production also differs among AD patients. The mean level of IL-10 among PBLs with complete resistance was 247.99 pq/ml, whereas in those with partial or lack of resistance it was 69.27 pq/ml (4 times less). However, the large spread of data probably caused the results to be scarce but beyond statistical significance. We did not observe any differences in spontaneous IFN-γ production among PBLs of AD patients. All data are presented in Fig. 2.

Similarly, statistically significant differences in VSV-induced TNF-α, and IL-1β production by PBLs of AD patients were observed. It was noticed that the level of TNF-α production by VSV-induced PBLs with complete resistance (mean level = 432.15 pq/ml) was more than 8 times higher compared to that of PBLs with partial/deficiency of resistance (mean level = 53.94 pq/ml). The effect size was medium on Cohen’s statistical effect size scale for both TNF-α, *r*_effect size_ = 0.520, and IL-1β, *r*_effect size_ = 0.395. Surprisingly, no differences were detected in VSV-induced IFN-γ production by PBLs depending on the PBLs resistance/innate immunity.

Among PBLs from AD patients, all cells, regardless of resistance to VSV infection/level of innate immunity, have responded with increased TNF-α production after VSV infection. Similarly, higher amounts of IL-1β after VSV infection were observed but only among PBLs with partial/deficiency of resistance. PBLs with complete resistance produced a comparable amount of IL-β spontaneously and after VSV infection. It seemed that generally, PBLs from AD patients with complete resistance to VSV infection produced large amounts of IL-1β, and VSV induction was not able to increase it more. Reverse effect was noticed for anti-inflammatory IL-10, where leukocytes obtained from all patients were characterized with a lower level of IL-10 production after VSV infection.

### PRP treatment decreased cytokine production by PBLs of AD patients

**Fig. 3:**
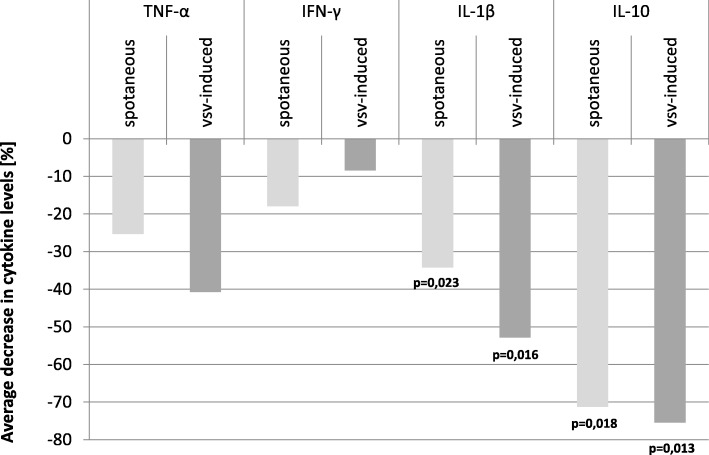
General average level of spontaneous and VSV-induced cytokine production by PBLs of AD patients after PRP treatment (*t* = 1). Freshly isolated PBLs (1 × 10^6^/ml) from blood samples of AD patients after PRP treatment were divided into two groups. One was cultured uninfected and second was infected with VSV (10^2^ TCID_50_/ml). After 40 min of VSV adsorption at RT, PBLs were washed with culture medium. All cells were next incubated at 37 °C for 24 h. Samples of medium above cultured uninfected (spontaneous cytokine production) and VSV-infected PBLs were collected for cytokine measurement with ELISA. Results has been analyzed with the Student *t* test for pairs

Next, we investigated the effect of PRP treatment on spontaneous and VSV-induced cytokine production by PBLs of AD patients. Although we observed a great increase in PBLs resistance/innate immunity after PRP treatment, there was no direct correlation of this effect with changes in investigated cytokine production (Table 3). However, what is noteworthy finding, generally after 4 weeks of PRP treatment, the average level of measured pro- and anti-inflammatory cytokine production by PBLs of all AD patients decreased. The most pronounced effect was observed for IL-1β (respectively spontaneous production decrease by 34.2%, VSV- induced by over 52.8%) and for IL-10 (respectively spontaneous production decrease by 71.2%, VSV-induced by 75.4%). Data are collected in Table 3. The same tendency was observed for TNF-α and IFN-γ, i.e., spontaneous TNF-α production by PBLs decreased by 25.3% and VSV- induced by 40.7%, and spontaneous IFN-γ production by PBLs decreased by 17.9% and VSV- induced by 8.4%. Lack of statistically significant results for TNF-α and IFN-γ production were due to a low number of subjects included into the study compared to high between-individual variability of this cytokine among the population. As a result, the test has too low power to recognize the observed difference as significant (power estimated in this case was 0.145 and 0.299 in case of spontaneous and VSV-induced samples, respectively). It is worth to mention that even if the observed difference would be statistically significant in case of a higher sample size, the value of this difference remains unchanged and smaller compared to IL-1β and IL-10. The same conclusion applies to IFN-γ, where a potential effect of the PRP is even smaller (− 17.9% and − 8.4% in case of spontaneous and VSV-induced production, respectively). Reduction of cytokine production by all investigated PBLs is shown in Fig. 3. There were no differences in cytokine production depending on the MMS score.

**Table 3:** Spontaneous and VSV-induced cytokine production by PBLs of AD patients (*n* = 20) after PRP treatment. General impact and data grouped by MMSE score are present. Means of ratio of the cytokine levels (before/after PRP treatment) are presented for all levels of the MMSE score. Virus effect was considered as the ratio of VSV induced and spontaneous. ANOVA linear contrast was used to test relationship between MMSE and virus effect (*F*-test). PRP effect was tested with Student *t* test

MMSE		*n*	TNF-α		IFN-γ		IL-1β		IL-10	
Range	Mean		Spontaneous	VSV- induced	Spontaneous	VSV- induced	Spontaneous	VSV- induced	Spontaneous	VSV- induced
23–22	22.2	6	0.320	0.161	0.817	0.989	0.729	0.691	0.131	0.213
21–19	19.6	5	1.181	2.548	0.766	1.123	0.794	0.935	1.091	0.868
18–14	15.2	5	0.491	0.420	1.036	0.712	0.352	0.145	0.059	0.027
13–12	12.5	4	2.551	1.039	0.674	0.867	0.973	0.498	1.303	0.987
Total mean ratio after/before (regardless of MMSE)		20	0.747	0.593	0.821	0.916	0.658	0.472	0.288	0.246
Mean change [%] after PRP treatment			− 25.3%	− 40.7	− 17.9	− 8.4	− 34.2	− 52.8	− 71.2	− 75.4
CI95% for the mean change	Lower		− 71.20%	− 81.6	− 33.8	− 25.5	− 54.8	− 76	− 89.8	− 91.4
	Upper		102.60%	102.60	3.30	10.10	− 5.40	− 14.50	− 16.90	− 22.80
*F*-test for MMSE effect H_0_: VSV effect independent of MMSE H_1_: linear relationship			0.179		0.293		2.794		0.695	
*p* value for *F*-test			0.678		0.596		0.114		0.417	
*r*_effect size_^*^ (MMSE effect size)			0.105		0.134		0.386		0.204	
Student *t* test for PRP effect H_0_: total mean ratio ≥ 1 H_1_: total mean ratio < 1			− 0.574	− 0.819	− 1.687	− 0.862	− 2.126	− 2.322	− 2.247	− 2.423
*p* value for the *t* test			0.2862	0.2114	0.054	0.1996	*0.0234* ^**^	*0.0158* ^**^	*0.0184* ^**^	*0.0128* ^**^
Power of the *t* test			0.145	0.299	0.191	0.092	0.685	0.918	0.915	0.943

^*^r_effect size_ means the correlation coefficient between ratio of VSV- induced and spontaneous and MMSE means both non-contrast between-group variation and the within-group variation are incorporated; ** statistically significant

### Influence of PRP on the distribution of PBLs’ subpopulations

Based on the aforementioned results, it was interesting to specify the potential effect of PRP on the distribution of PBLs’ subpopulations. First, the influence of PRP on PBLs proliferation was measured (Additional file 1). Five independent experiments with 8 repetitions each were performed. We observed that PRP does not affect PBLs proliferation. From Table 4, we can see that the average absorbance for PBLs culture (control) is 0.303 and the average absorbance for PBLs after PRP treatment is 0.311 (*p* = 0.592). This relevant observation showed that PRP did not exhibit a pro-proliferative activity (Additional file 2: Figure S1).

**Table 4:** Distribution of leukocytes with basic statistics at time *T* = 0 (PBLs without PRP treatment, control), after 24 h of PBLs culture without PRP treatment (control) and after 24 h of PBLs culture with PRP 120 μg/ml (*N* = 21). Difference = PRP_24h – Control_24h. For ANOVA, see Additional file 2: Table S1. Additionally, the influence of PRP on PBLs proliferation was reported as the mean of 5 means, where every single mean was computed based on *n* = 8 independent measurements (*N* = 40)

Cell	Statistics	*T* = 0 PBLs	*T* = 24 h PBLs	*T* = 24 h PBLs + PRP	Difference (*T* = 24 h)
Leukocytes (PBLs)	Mean	6.57	6.40	6.71	0.31
	SD	2.68	3.42	2.82	1.03
Lymphocytes	Mean	1.61	2.53	2.23	− 0.30
	SD	0.65	1.13	0.79	0.58
	%	24.93	44.09	36.06	− 8.03
Monocytes	Mean	0.3	1.13	0.77	− 0.36
	SD	0.12	0.60	0.21	0.57
	%	4.8	18.47	12.31	− 6.16
Granulocytes	Mean	4.66	2.70	3.71	1.01
	SD	2.1	2.40	2.53	0.36
	%	70.27	37.44	51.63	14.19
Changes in distribution of PBLs’ subpopulations after PRP treatment^*^			*F*_2; 12_ = 20.95; *p* = 0.000122		
Proliferation	Statistics		PBLs	PBLs+PRP	Difference
	Mean		0.303	0.311	0.0078
	SD		0.0352	0.0549	0.0299^**^
Influence of PRP on PBLs proliferation			*F*_1; 4_ = 0.339; *p* = 0.592		

*SD* standard deviation. ^*^For more details, see Additional file 2: Table S1

^**^This is standard deviation of five mean differences (not difference between two SD)

**Fig. 4:**
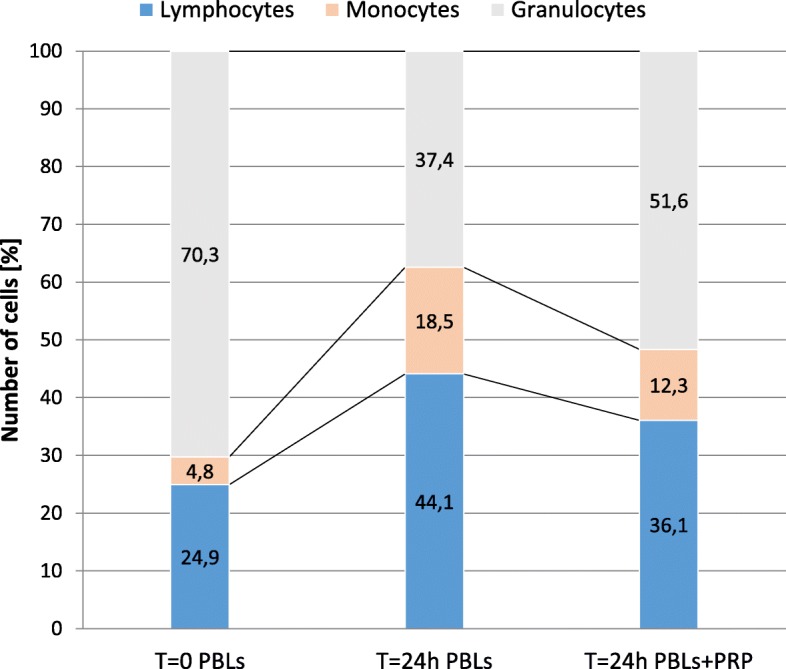
Distribution of PBLs’ subpopulations at time *T* = 0 (PBLs without PRP treatment, control), after 24 h of PBLs culture without PRP treatment (control) and after 24 h of PBLs culture with PRP 120 μg/ml. For ANOVA of changes between PBLs *T* = 24 h and PBLs+PRP *T* = 24 h, see Table 4

It was showed, however, a significant influence of PRP on the distribution of PBLs’ subpopulations. Seven independent experiments with blood samples from AD patients were performed. From Table 4, we can see that PRP did not affect leukocytes’ subsets in the same way (*n* = 7, *p* = 0.000122). We noticed that the treatment decreased the number of lymphocytes and monocytes (about 8 and 6 percentage points, respectively) and increased the number of granulocytes by an average of 14 percentage points. There was no difference between lymphocytes and monocytes after PRP treatment; the degree of reduction was the same (*p* = 0.8158) and differed from an increase of the number of granulocytes (*p* = 0.00003). Results are presented in Fig. 4.

## Discussion

Despite decades of research, the causes of AD still remain unclear. The psycho-neuroinflammatory theory suggests that inflammation may play a key role in psychiatric disorders in the elderly. Recent scientific reports supply many data that emphasize the central role of impaired immune response and inflammation in AD, with the influence of cytokines in the regulation of neurodegenerative processes [25]. AD is considered as a systemic disease that affects inflammatory reactions in the brain as well as in the periphery, and it is suggested that increased peripheral inflammation leads to more neurodegeneration [5]. It is postulated that peripheral immune cells infiltrating the brain are an additional source of inflammatory markers and indicate systemic inflammatory processes which may be related directly or indirectly to AD risk [26, 27]. Chronic inflammatory response in the periphery, increasing with age, and aged-related infections may also contribute to the disease progression with pathological changes seen clinically and lead to the development of overt clinical AD. It is pointed that the activation status of peripheral innate immune cells should be considered as an early biomarker of brain pathology. Therefore, modulation of these cells might be also a promising tool for modifying AD progression [28]. However, there are many conflicting studies about the association between markers of inflammation and risk of dementia [29]. Recently published systematic review and meta-analysis evaluated the peripheral levels of pro-inflammatory markers including IL-1β, IL-6, TNF-α, and CRP between the elderly with AD and controls without any psychiatric disorder. It was showed that the elderly with AD did not have higher peripheral inflammatory markers [30]. Nevertheless, there are some studies on the peripheral IL-6 that may be a useful biological marker to correlate with the severity of cognitive impairment [31]. It is possible that this inconsistency may be attributed to the complex and overlapping relationship between inflammatory markers and AD. Moreover, difference in researchers’ findings might be related to study design, characteristics of participants, or incorrect data analysis. Nonetheless, we can investigate peripheral immune cell activation and response and then conclude on their condition and capacity to generate inflammatory response. Inflammatory pathways are altered in the periphery in AD, and peripheral immune cell responses reflect inflammatory mechanisms better in comparison to the serum/plasma. They are displayed on the central nervous system (CNS) metabolites absorbed daily into the blood through the blood-brain barrier (BBB) as well as on other harmful stimuli including infections [32, 33]. Moreover, it is most likely that an increased level of inflammatory markers is not constantly maintained in the plasma of patients with AD.

As the first line of defense of the body against different environmental hazards, innate immunity plays a central role in many inflammatory and infectious diseases. It is believed that peripheral immune cell activation participates in AD pathogenesis [28]. In the current preliminary study, we analyzed two reactions of innate immunity, i.e., ex vivo PBLs resistance to viral infection, and cytokine production by PBLs from AD patients before and after proline-rich polypeptide complex (PRP) treatment. Needless to say that PRP possesses immunoregulatory properties and is advocated as a nutritional countermeasure to immune dysfunction. For that reason, we assumed that PRP treatment might have a beneficial effect on the activation of peripheral innate immune cells in AD patients, and we investigated its influence on the reactions of innate immune response. Firstly, we observed that investigated PBLs were characterized with different susceptibility to VSV infection. VSV has replicated freely in most of PBLs reaching the titer of 3–4 and more logarithms. This confirmed our latest results that unspecific PBLs resistance is reduced during aging and suggestions that PBLs of AD patients are high susceptible to VSV infection compared to healthy elderly [10, 23]. However, the following investigations allowed us to obtain more interesting data on the degree of PBLs resistance in relation to severity of AD. Patients diagnosed with serious AD were characterized with higher sensitivity of PBLs to VSV infection, while patients with moderate AD showed partial resistance to VSV. Complete resistance of PBLs was noticed among patients with mild AD. Possibly, PBLs resistance should be considered as a promising marker of AD progression.

The next step was to study the cytokine profile of cultured PBLs resistant and susceptible to VSV infection. Statistically significant differences in cytokine production were demonstrated, showing that PBLs which were completely resistant to VSV infection produced higher levels of pro- and anti-inflammatory cytokines: TNF-α, IL-1β, and Il-10 compared to sensitive PBLs. The most meaningful were the results of cultured PBLs (spontaneous cytokine production). Moreover, we noticed that average levels of mentioned cytokines were much higher compared to healthy individuals, which is in agreement with our earlier studies [10, 11, 23]. These results clearly indicated pronounced pro-inflammatory features of PBLs of AD patients. Similarly, the study of Ciaramella et al. [34] showed that AD-linked dysregulation of immune mechanisms may lead to dendritic cell-mediated over-activation of inflammation and impaired antigen presentation. The authors showed pro-inflammatory status of MDDCs as well as monocytes (MDDC precursors) from AD patients in comparison to cells from healthy donors. The observed PBLs resistance to viral infection is related to the infection of specific leukocyte subpopulations, and therefore, the observed level of immune response of PBLs of AD patients may be related to changes in the distribution of leukocytes’ subsets and function and/or impaired immune response to harmful stimuli that take place during senescence of the immune system. Another attribute of innate immunity is dependence of the resistance on small amounts of cytokines: interferons and tumor necrosis factor. Based on this, the resistance was found to be individually differentiated and depended on human age [10]. More recent studies of Le Page et al. [35] also have confirmed an impaired response of peripheral immune cells associated with AD development. They showed differentially altered capacity of polymorphonuclear neutrophils (PMNs) in peripheral blood from patients with amnestic mild cognitive impairment and patients with mild AD in response to pathological aggression. However, in those studies, the authors showed that PMNs produced very low inflammatory cytokines (TNF-α, IL-6, IL-1β, IL-12p70) and chemokines in response to LPS stimulation. Additionally, Chen et al. [36] demonstrated alterations in quantity and quality of blood cells, e.g., decreased level of lymphocytes and basophils, which suggested that this may contribute to AD progression. Similar results were obtained by Richartz-Salzburger et al. [37].

**Fig. 5:**
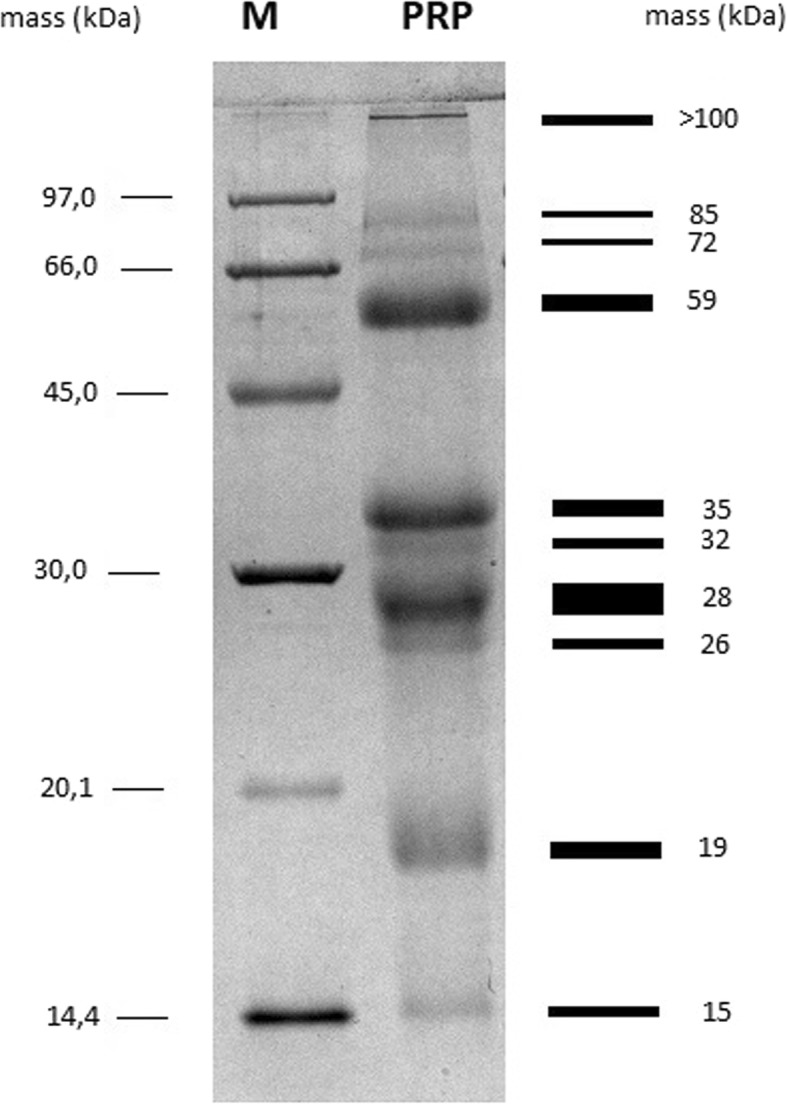
SDS-PAGE analysis of PRP preparation. PRP were loaded in “PRP” lane of 12.5% polyacrylamide gel. Next, the gel was stained with Coomassie brilliant blue and visualized by using Bio-Rad Gel Doc™ XR+. Line “M” represents protein molecular mass standard

Several research and clinical studies revealed that PRP provides many therapeutic properties from immunomodulatory (in vitro) therapy to effectively enhance pro-cognitive functions in animal models and AD patients [21]. PRP investigated in this study is a dietary supplement commonly recommended to AD patients to improve cognitive functions. It is a complex of peptides with molecular masses from approximately 15 to over 100 kDa. Most of them are proteins with a mass molecule of approximately 19, 28, 35, and 59 kDa. Less-visible bands represent proteins with a mass molecule of about 15, 26, 32, 72, 85, and > 100 kDa. Protein content and molecular weight of PRP preparation are shown in Fig. 5.

Until now, no examinations evaluated influence of PRP treatment on innate immune reactions in AD patients. Our study sheds light on the need for immunomodulatory therapy in AD and indicates potential use of PRP to modulate an innate immune response. PRP tablets that were orally administrated to AD patients in this study are coated with a gastric juice-resistant envelope. This makes the tablets disintegrate and release substances only in the small intestine, protecting the active PRP from the digestive enzymes. Therefore, PRP is absorbed directly from the small intestine preserving its biological activity. It was noticed that PRP from the colostrum is absorbed by the gut-associated lymphoid tissue (GALT) where it may influence the immune system cells [38]. PRP immunoregulatory activity was demonstrated already for certain sequences of the polypeptide as well as for synthetic analogs of active PRP fragments. When PRP was subjected to chymotrypsin digestion and separated by gel filtration, three fractions were obtained. All of PRP fractions retained their immunological activity, especially the shortest one, a nonapeptide (Val-Glu-Ser-Tyr-Val-Pro-Leu-Phe-Pro), which showed the full spectrum of the biological effects of PRP [39, 40].

The study of the effect of PRP from bovine colostrum in AD patients provided interesting results, pointing its possible beneficial, immunomodulatory effects in AD therapy. After 4 weeks of treatment with PRP, we observed an increase PBLs resistance to VSV infection ex vivo in almost all patients or maintain already high PBLs resistance. In general, we observed a compensation of investigated mechanism of innate immunity—PBLs resistance—to an equal level in all patients after PRP treatment. We speculated that this phenomenon might be related to cytokine response by PBLs due to knowing of immune and overall health benefit activities of mammalian colostrum. PRP is not species-specific; however, according to some authors, PRP may display different immune activities depending on the source and method of isolation [41].

An immunomodulatory activity of PRP from ovine colostrum (Colostrinin) was shown in several in vitro studies [21, 42]. It was considered as a modest cytokine inducer, IFN-γ and TNF-α, in human PBLs and whole blood cell culture (WBC) [43] as well as IL-6 and IL-10 [40]. Moreover, Colostrinin was investigated for pro-cognitive properties. PRP-treated volunteers showed signs of improvement in mood and cognitive abilities. Respectable studies have been carried out on the potential efficacy of Colostrinin in AD patients. First clinical trials with Colostrinin showed improvement and stabilization of the health status of AD patients [44–46]. Colostrinin has also shown an antiviral activity in mouse model of resident peritoneal cells [20]. In our study, an improvement of PBLs resistance after PRP from bovine colostrum treatment was not related to changes in any of measured pro- and anti-inflammatory cytokine production. The most important general decrease in TNF-α, IL-1β, IL-10, and IFN-γ production by PBLs of AD patients after PRP treatment was noticed. Initially, we thought that it might be associated with INF-α production, in which large amounts secreted by PBLs after VSV infection were demonstrated [11]. Nevertheless, in vitro studies with PRP did not confirm an inhibition of VSV replication in PBLs (data not shown); thus, we concluded that intracellular activation of IFN-α, the main antiviral agent, pathway was not supported by this preparation. It is worth to mention that such anti-inflammatory properties of PRP from ovine colostrum, i.e., inhibition of ROS, NO, and pro-inflammatory cytokine production as well as activation effect on glutathione peroxidase (GSHPx) and glutathione reductase (GSSGR) by human peripheral blood mononuclear cells (PBMC) after LPS stimulation, were observed earlier [47, 48]. Given the in vitro results and lack of significant influence of PRP on IFN-γ production by PBLs in the current study, we suspected that this preparation does not activate IFN signaling. These results prompted us to study the potential effect of PRP on the distribution of leukocyte subpopulations as a possible mechanism of the observed pro- and anti-inflammatory cytokine blanking and innate immune improvement. Indeed, PRP changed the content of particular cell fractions by increasing granulocyte (mostly neutrophils) survival and decreasing propagation of monocytes and lymphocytes. PRP did not exhibit a pro-proliferative activity. It is most likely that PRP treatment increased the resistance/immune response of PBLs and general decrease of cytokine response by several possible ways. First, as the only modest IFN inducer, PRP may influence the intracellular signaling pathways of other cytokines and/or chemokines, which is not investigated in this study. Nevertheless, the general decrease of cytokine production and subduing the excessively developed inflammatory response was noticed after PRP therapy. Possibly, by beneficial effect on neutrophils, PRP induced other than IFNs mechanisms of antiviral response. Neutrophils are the first immune cells engaged to halt a viral infection [49, 50]. Various mechanisms to restrict viral infections may be used by neutrophils, such as phagocytosis, production of cytokines, and antimicrobial agents like ROS, neutrophil extracellular traps (NETs), or direct defensin targeting of viral envelopes, glycoproteins, and capsids as well as inhibition of viral fusion and post-entry neutralization [49, 51]. PRP has been shown to decrease ROS production [52]. It was previously noted, however, that α-defensin HNP-1 can directly inactivate VSV in vitro [53]. Taken together, change in the distribution of PBLs’ subpopulation by a decrease in monocyte promotion, the main target cell for VSV, and increase in importance of neutrophils in innate immune response might at least partially explain the beneficial effect of PRP treatment in AD. Studies with much higher numbers of patients and measuring of IFNs as well as a large spectrum of other pro- and anti-inflammatory cytokines and other innate immune mechanisms (phagocytosis, antimicrobial protein activity) are necessary to pinpoint the observed beneficial effect of PRP on investigated innate immune mechanisms in AD patients.

This paper elucidates highly important and interesting observations, despite the lack of external controls. Nevertheless, the absence of age-matched healthy subjects as well as a placebo control group should not be considered as our negligence in this study. Firstly, a healthy control group was not essential as this is a before-after study, and secondly, the observed results for AD patients still remain correct and well-founded. The use of placebos is a standard control component of most clinical trials; however, it does not guarantee that it is more effective, or even as effective as an examined agent. It is difficult to conceive that the observed disturbance in blood population distribution after PRP treatment was likely due to a placebo effect. However, our preliminary study results should be followed by larger randomized, placebo-controlled clinical trials in the future.

Nevertheless, still the remaining question is what could be the clinical consequences of the observed results? PRP-induced decreased cytokine production by PBLs may be very important because there are strong evidence for the concept that neuroinflammatory processes are a major risk factor for AD. Neuroinflammation induces inflammatory mediators like cytokines which work to perpetuate the inflammatory cycle, activating microglia, promoting their proliferation, and resulting in further release of inflammatory factors. In this context, it is worth to add that epidemiological studies indicate that non-steroidal anti-inflammatory drugs (NSAIDs) reduce the risk of AD, providing evidence that inflammation mechanisms play a role in this disease. We know that risk for conversion from mild cognitive impairment to the dementia stage of AD is increased in patients with elevated concentrations of the pro-inflammatory cytokine TNF-α and decreased concentrations of anti-inflammatory transforming growth factor beta (TGF-β) in the CSF. There is a strong need for mechanisms explaining the development of AD to be proposed with some immediate suggestions aimed at treatment interventions. Following this trend, the role of innate and acquired immunity in AD is under intensive study. We found that some therapeutics like PRP seemed to increase the resistance of PBLs, which suggested a positive influence on patients suffering with AD. It is widely accepted that for the development and regulation of innate immunity, many agents (receptors, signaling molecules, cytokines, adhesion molecules, etc.) and mechanisms are involved. Therefore, therapeutic strategies which we proposed using PRP, which induce immunological response, may be viewed as potential treatment. Theoretically, such treatment can decrease infiltration of the rims of senile plaques by immune cells and also lead to a diminution in the release of cytokines and other soluble factors. In general, recognition that modification of the immune system contributes to pathogenesis of AD opens potential routes to delay its onset and progression. This interesting field of research will serve to identify novel therapeutic targets that may finally lead to a long-awaited victory in the war on AD. According to our opinion, PRP treatment provides an exciting and new opportunity to look into neuroinflammatory processes in AD and shed a new light on the complex interaction within CNS.

## Conclusions

Emerging data highlight the role of innate immunity molecules such as complement, class I major histocompatibility complex, and Toll-like receptor system or pro- and anti-inflammatory cytokines in pathomechanism of neurodegeneration of Alzheimer’s type. The access of the peripheral immune system to the CNS is restricted and tightly controlled, but the CNS is quite capable of dynamic inflammatory and immune responses to a wide range of attacks and insults. Some factors such infections, trauma, toxins, stroke, and other stimuli have been reported to generate immediate and short-lived activation of the innate immune system within the CNS. This sensitive neuroinflammatory response is mediated by the activation of native microglial, which results in a phagocytic phenotype and release of inflammatory mediators such as chemokines and cytokines. Our findings supported for the first time that patients with Alzheimer’s have a reduced level or deficiency in innate immunity of PBLs that correlated with the severity of the disease. PRP treatment increased the level of innate immunity, measured with PBLs resistance to viral infection, and showed a general decrease in pro-inflammatory cytokines, thereby reducing inflammatory response. Future research on the regulation of inflammatory response and their influence on neurodegeneration could provide significant improvement of repertoire of early diagnostic biomarkers and will also open a new adventure for more effective treatment of neurodegeneration like Alzheimer’s disease.

## Additional files

Additional file 1: Data set. (XLSX 18 kb)

Additional file 2: **Figure S1.** Mean level of absorbance as proliferation measure. Every point is mean of *n* = 8 measurements for one experiment - PBLs with and without PRP treatment. Dot line with slope of 45° represents situation with no change. ANOVA of randomized block design showed that observed points are randomly located around this line (*p* = 0.592). There is no change in PBLs proliferation after PRP treatment. **Table S1.** ANOVA for PRP treatment after 24 h according to PBLs distribution in randomized block design. Changes in number of cells were a response, i.e., difference between PRP treatment after 24 h and without PRP treatment after 24 h. Sum of squares for type of cell was divided into two orthogonal contrasts: (c1) difference between mean changes in subpopulations of lymphocytes and monocytes and (c2) difference between lymphocytes and monocytes (pooled) and granulocytes. (DOCX 45 kb)

## Data Availability

All data generated or analyzed during this study are included in supplementary information files.

## Abbreviations

AD: Alzheimer’s disease
BBB: Blood-brain barrier
BMI: Body mass index
CNS: Central nervous system
CRP: C-reactive protein
CT: Computer tomography
EEG: Electroencephalographic
ELISA: Enzyme-linked immunosorbent assay
FBS: Fetal bovine serum
GALT: Gut-associated lymphoid tissue
GSHPx: Glutathione peroxidase
GSSGR: Glutathione reductase
IFN-γ: Interferon gamma
IL-10: Interleukin 10
IL-1β: Interleukin 1 beta
ISGs: IFN-stimulated genes
MDDCs: Monocyte-derived dendritic cells
MMSE: Mini-Mental State Examination
MRI: Magnetic resonance imaging
MTT: 3-(4,5-Dimethylthiazol-2-yl)-2,5-diphenyltetrazolium bromide
NETs: Neutrophil extracellular traps
NSAIDs: Non-steroidal anti-inflammatory drugs
PBLs: Peripheral blood leukocytes
PBMC: Peripheral blood mononuclear cells
PMNs: Polymorphonuclear neutrophils
PRP: Proline-rich polypeptide complex
RLRs: Retinoic acid-inducible gene I (RIG-I)-like receptors
TCID_50_: Tissue culture infectious dose observed in 50% cells
TGF-β: Transforming growth factor beta
TNF-α: Tumor necrosis factor alpha
VSV: Vesicular stomatitis virus
WBC: Whole blood culture
