# Supplementary material for: New therapeutic targeting of Alzheimer’s disease with the potential use of proline-rich polypeptide complex to modulate an innate immune response - preliminary study
Source: J Neuroinflammation. 2019 Jul 5;16:137. doi: 10.1186/s12974-019-1520-6 (PMC6612126; doi:10.1186/s12974-019-1520-6)
Supplement: Supplementary file 2 — Figure S1. Mean level of absorbance as proliferation measure. Every point is mean of n = 8 measurements for one experiment - PBLs with and without PRP treatment. Dot line with slope of 45° represents situation with no change. ANOVA of randomized block design showed that observed points are randomly located around this line (p = 0.592). There is no change in PBLs proliferation after PRP treatment. Table S1. ANOVA for PRP treatment after 24 h according to PBLs distribution in randomized block design. Changes in number of cells were a response, i.e., difference between PRP treatment after 24 h and without PRP treatment after 24 h. Sum of squares for type of cell was divided into two orthogonal contrasts: (c1) difference between mean changes in subpopulations of lymphocytes and monocytes and (c2) difference between lymphocytes and monocytes (pooled) and granulocytes. (DOCX 45 kb) [file 12974_2019_1520_MOESM2_ESM.docx]

**Figure S1.** Mean level of absorbance as proliferation measure. Every point is mean of n=8 measurements for one experiment - PBLs with and without PRP treatment. Dot line with slope of 45 degree represents situation with no change. ANOVA of randomized blocks design showed that observed points are randomly located around this line (p=0.592). There is no change in PBLs proliferation after PRP treatment.

**
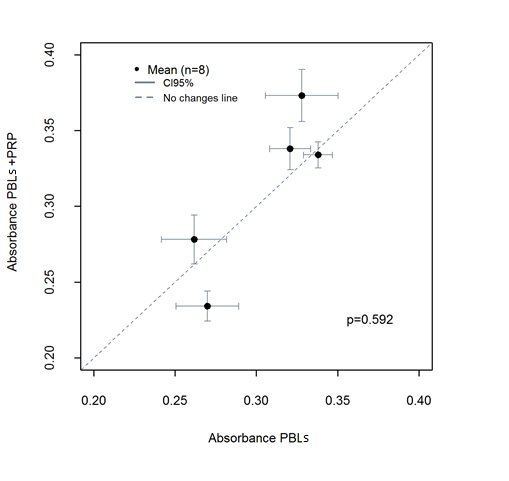
**

**Table S1.** ANOVA for PRP treatment after 24h according to PBLs distribution in randomized blocks design. Changes in number of cells was response, i.e. difference between PRP treatment after 24h and without PRP treatment after 24h. Sum of squares for type of cell was divided into two orthogonal contrasts: c1) difference between mean changes in subpopulations of lymphocytes and monocytes and c2) difference between lymphocytes and monocytes (pooled) and granulocytes.

| **Source of variation** | **Degrees**  **of freedom** | **Sum**  **of squares** | **Mean**  **Square** | **F-test** | **p-value** |
| --- | --- | --- | --- | --- | --- |
| Blood sample/donor (blocks) | 6 | 2.292 | 0.3821 | - | - |
| **Type of cell** | 2 | 8.426 | 4.213 | 20.95 | 0.000122 |
| c1) Lymphocytes vs Monocytes | 1 | 0.0114 | 0.0114 | 0.057 | 0.8158 |
| c2) (Lymphocytes+Monocytes)  vs Grannulocytes | 1 | 8.415 | 8.415 | 41.866 | 0.000031 |
| Residuals | 12 | 2.413 | 0.201 |  |  |
| Homogeneity of variances | Fligner-Killeen test $\chi_{df=2}^{2}=0.838;p=0.6577$ | | | | |
